# Innovative Imaging Techniques for Advancing Cancer Diagnosis and Treatment

**DOI:** 10.3390/cancers16142607

**Published:** 2024-07-22

**Authors:** Tianyuan Wang, Yicheng Ni, Li Liu

**Affiliations:** 1 Department of Radiology, The University of Texas Southwestern Medical Center, Dallas, TX 75390, USA; tianyuan.wang@utsouthwestern.edu; 2 Department of Radiology, Zhongda Hospital, Southeast University, Nanjing 210009, China

## 1. Introduction

Traditional oncology image-analysis, using modalities such as echography, X-ray, CT, and MRI, has historically relied on human-defined features to interpret and assess clinical images [1]. Early diagnostic decision support systems were constrained by low precision, leading to more unnecessary biopsies [2]. The lack of advanced imaging methods impeded the efforts to predict therapy response and to personalize treatments. Recent breakthroughs in oncologic imaging have transformed cancer diagnosis, treatment planning and monitoring, providing clinicians with remarkable insights into tumor characteristics and responses to therapies across various cancer types and treatment stages. For instance, dual-modality imaging, such as PET/CT (commercialized in 2001) and SPECT/CT (commercialized in 2004), combine anatomical, metabolic and functional information for more accurate disease assessment. These technologies have enhanced medical imaging by allowing for more accurate tumor localization, staging, and treatment response monitoring, ultimately improving patient outcomes, especially in oncology [3]. The emerging and continual advancement of molecular imaging offers early cancer detection by visualizing molecular and cellular processes, aiding in timely intervention and prevention of advanced disease stages like metastases. These technological breakthroughs provide deeper insights into pathophysiologic processes, making it a valuable tool for improving patient care [4]. There have been several notable developments in molecular imaging, although mostly unready yet at clinical scales. The super-resolution fluorescence microscopy allows nanoscale visualization of cellular processes [5]. DNA-based point accumulation in nanoscale topography (DNA-PAINT) achieves sub-20 nm resolution by leveraging transient DNA binding events [6]. Photoacoustics represents the newest modality available for probing dynamic vascular activity in vivo, offering advantages such as non-invasiveness and the ability to image deep tissues [7]. Over the last decade, artificial intelligence (AI) has seen significant growth, particularly in radiation oncology, where advanced computational methods aim to deliver personalized cares with high diagnostic and therapeutic precision [8]. The abundance of imaging data, coupled with advances in machine learning and deep learning, has enabled the discovery of hidden biomarkers and quantitative features in medical images [9]. So far, five original research papers and one review article have been published in the special issue, highlighting the benefit of recent imaging techniques in the following aspects of oncology.

## 2. Diagnosis and Detection

Stimulated Raman scattering (SRS) is a fast, label-free imaging method that offers high-resolution histologic images by using the molecular vibrational properties of chemical bonds in macromolecules such as lipids, proteins, and nucleic acids [10]. It was developed by Freudiger Xie and colleagues in 2008 [11] and has since been used in biomedical and brain tumor imaging. Recent advances in fiber laser technology have led to the first clinical SRS microscope for fresh tissue imaging in surgical pathology and surgical oncology [12]. Andreas Weber et al. explored the application of deep learning in classifying oral squamous cell carcinoma (OSCC) and non-malignant tissue types using Stimulated Raman Histology (SRH). They compared the performance of their deep learning model trained on SRH images with the original images obtained from SRS. The deep learning model trained on SRH images shows potential for effective tissue identification during oral cancer surgery, thereby expediting decision-making.

Dynamic contrast-enhanced (DCE) MRI offered flexible perfusion imaging and rapid qualitative assessment and quantitative measures of intrinsic perfusion and permeability parameters [13]. Studies have explored the potential of DCE MRI to enhance cancer detection, characterization, response assessment, and planning for radiation therapy [14]. However, quantitative DCE MRI’s clinical use remains limited outside of trials. Chad A. Arledge et al. investigated the utility of a deep learning approach in assessing vascular permeability changes in brain metastasis post-whole-brain radiotherapy. They employed convolutional neural networks (CNN) trained on DCE MRI data of glioblastoma mice and transfer the learning to whole brain radiation therapy-treated brain metastasis mice. The study demonstrates efficient and accurate evaluation of vascular permeability in brain metastasis.

## 3. Prediction and Prognosis

Radiomics involves extracting high-dimensional data from multimodality oncology images and has been applied in oncology to enhance diagnosis and prognosis, aiming to enable precision medicine [15]. Two CT-based Radiomic studies are included in this special issue. In Chao Li et al.’s study on locally advanced rectal cancer (LARC), a radiomics approach was used to extract quantitative imaging features from pretreatment contrast-enhanced CT scans. They developed and validated a radiomics nomogram that combined the planning CT-based Radscore with the pretreatment T stage for individualized prediction of pathological complete response (pCR) before neoadjuvant chemoradiotherapy. Using support vector machine (SVM), they selected radiomics predictors and achieved robust prognostic performance for pCR. The SVM method demonstrated superiority over other classifiers, effectively handling high-dimensional data and small datasets. Despite some limitations, the study suggests the SVM-based nomogram is a promising non-invasive tool for predicting treatment outcomes in LARC patients. Another study from Huei-Yi Tsai et al. demonstrates that a combination of machine learning models using radiomics features from pretreatment contrast-enhanced CT and clinical data could accurately predict pCR in breast cancer patients. The approach proved more cost-effective and efficient compared to other imaging modalities like MRI or PET/CT. By incorporating more categories of textural features and ensuring a standardized feature extraction process, the models’ performance was improved, highlighting the benefits of integrating clinical and CT-based radiomics features for predicting pCR in breast cancer.

## 4. Treatment Planning and Monitoring

Vascular disrupting agents (VDAs) have emerged as a promising class of targeted cancer therapies that exploit the unique features of the tumor microenvironment. VDAs selectively damage those tumoral micro-vessels, effectively starving the tumor of oxygen and nutrients, leading to extensive necrosis [16]. The rapid development of VDAs over the past decade has integrated advanced preclinical imaging and evaluation methods to predict clinical efficacy, making them a key focus for future anticancer therapies. However, the heterogeneity of tumors and the complex interaction between tumor vasculature and therapeutic agents pose challenges to the successful application of VDAs. Shuncong Wang et al. revealed the notable differences in the effects of VDAs on intracranial and extracranial tumors, as assessed through multiparametric imaging data. They showed superior pre-treatment blood perfusion and stronger vascular shutdown in extracranial tumors, indicating improved drug penetration and therapeutic outcomes. These findings underscore the crucial role of imaging modalities, particularly quantitative perfusion-weighted imaging (PWI) parameters, in evaluating tumor response to VDAs and guiding patient selection for targeted therapies.

## 5. Surgical Guidance

The quest for complete tumor removal in hepatopancreatobiliary malignancies remains a pivotal goal to enhance patient survival and improve outcomes [17]. Tereza Husarova et al. stated that achieving oncological radicality is challenging due to vital vascular structures in the liver and pancreas, and difficulties distinguishing viable tumors from fibrosis in preoperative imaging after neoadjuvant therapy. In their review article, they introduced cutting-edge technologies in intraoperative imaging within hepatopancreatobiliary surgery. They focused on novel imaging modalities and targeted contrast agents that hold the potential to refine preoperative and intraoperative diagnostics. This review paper examines recent innovations in preclinical research and assesses their clinical implications and future perspectives, including the essential attributes of an ideal contrast agent. It provides valuable insights for healthcare professionals invested in the evolution of surgical practices to enhance oncological outcomes and patient care.

In conclusion, the studies featured in this special issue offer encouraging progress in the integration of deep learning and advanced imaging modalities for disease diagnostics and treatment. However, several challenges persist that could limit the applicability of findings across diverse groups and conditions. Some potential directions should be considered: (1) the use of small, homogeneous datasets may not fully capture the heterogeneity of patient populations and different disease subtypes, which can restrict the generalizability of models and limit their clinical utility. Future research should prioritize the expansion of datasets for training and validation to better account for variations in patient characteristics and to respond more effectively to different clinical scenarios; (2) exploring new imaging modalities and using AI techniques to handle imbalanced datasets and uncover subtle patterns in medical images can further enhance predictive accuracy and decision-making; and (3) despite the rapid development of new oncology technologies, ongoing research and development are necessary to refine existing technologies, combine them with new advances, and optimize their integration into clinical workflows. The collaboration between researchers, clinicians, and industry is crucial to address these challenges, translate preclinical innovations into effective clinical practice, and drive significant improvements in patient care and outcomes across a wide range of medical fields.
